# Atypical Presentation of an Internal Capsule Infarct With Isolated Acute Confusion: A Case Report

**DOI:** 10.7759/cureus.96119

**Published:** 2025-11-05

**Authors:** Morgan W Hayden, Karen Chouhan

**Affiliations:** 1 Internal Medicine, The Grange University Hospital, Cwmbran, GBR; 2 Stroke Medicine, The Grange University Hospital, Cwmbran, GBR

**Keywords:** acute confusion, atypical stroke presentation, confusion, internal capsule infarct, stroke

## Abstract

Stroke of the internal capsule characteristically presents with pure motor or sensory deficits from the disruption of the corticospinal and thalamocortical fibers. However, specific involvement of several subcortical pathways can result in cognitive rather than motor or sensory presentations. We report the case of a 70-year-old woman presenting with acute confusion and word-finding difficulty. Computed tomography (CT) of the brain was reported as negative for acute ischemia or hemorrhage, but magnetic resonance imaging (MRI) demonstrated an acute infarct involving the posterior limb of the left internal capsule with extension into the medial temporal lobe and caudate nucleus. The patient's cognition improved rapidly, and she was discharged on anticoagulation and secondary prevention for concurrently diagnosed atrial fibrillation. This case stresses that subcortical infarcts can produce isolated cognitive symptoms. Recognition of atypical presentations is essential, with early MRI facilitating timely diagnosis, leading to the correct management with anticoagulation, rate control, and secondary prevention.

## Introduction

Stroke remains the second most common cause of death worldwide [1]. Ischemic strokes account for up to 62% of strokes globally, with 25% of those strokes being lacunar [1]. Lacunar strokes refer to a subset of ischemic strokes that result from the occlusion of small, penetrating arteries that supply the thalamus, pons, basal ganglia, and internal capsule [2]. In lacunar infarction, the occlusion can be a result of atheromatous disease, lipohyalinosis, or embolic fragments from a cardiac source such as a ventricular thrombus or atrial fibrillation. There are numerous clinical manifestations of strokes resulting from occlusions to these small arteries; however, these lacunar syndromes most commonly present with a subset of symptoms such as pure motor hemiparesis, ataxic hemiparesis, pure sensory loss, dysarthria-clumsy hand, and sensory motor loss [1]. Lacunar infarcts infrequently result in higher cortical deficits, including memory, language, or attention. However, lacunar strokes to the internal capsule may rarely result in cortical dysfunction, including confusion and behavioral changes [1].

The internal capsule is an area supplied by these small penetrating arteries. It is a small white matter structure that conduits the cerebral cortex and subcortical nuclei, brainstem, and spinal cord. It comprises five main components: the anterior limb, genu, posterior limb, and retrolenticular and sublenticular parts [2]. The anterior limb, for example, contains frontopontine and anterior thalamic radiations involved in cognition and behavior. The genu transmits corticobulbar fibers controlling cranial motor function. The posterior limb conveys corticospinal and sensory pathways involved with voluntary movement and sensation [3]. Finally, the retrolenticular portion carries optic radiations, with the sublenticular part transmitting auditory fibers [2]. This wide array of conduits explains the variety and peculiarity of clinical syndromes that damage to various structures can produce.

We present an unusual case of isolated confusion resulting from a lacunar infarct affecting the left posterior internal capsule.

## Case presentation

**Figure 1:**
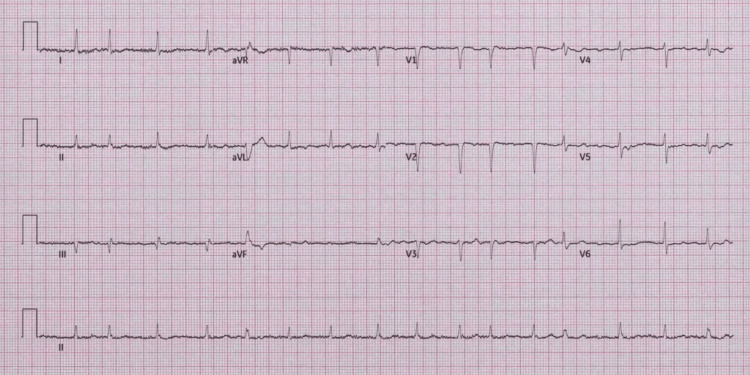
Electrocardiography showing rate-controlled atrial fibrillation

A 70-year-old right-handed woman, with no past medical history, presented to the emergency department with acute confusion and word-finding difficulty. Symptoms began two days after returning from a holiday, following a self-limiting episode of diarrhea. On examination, she was alert, disoriented, with fluent but circumlocutory speech, and not oriented to time, place, or person. Cognitive screening using the 4 A's Test (4AT) scored 1 out of 4, suggesting significant acute cognitive impairment. Of note, no dysarthria, facial asymmetry, motor weakness, or sensory deficit was identified. Crucially, the cognitive deficits and effect on language were not felt to represent receptive or expressive dysphasia. Cardiovascular examination revealed an irregular pulse, and electrocardiography confirmed new-onset, rate-controlled atrial fibrillation (Figure 1).

**Figure 2:**
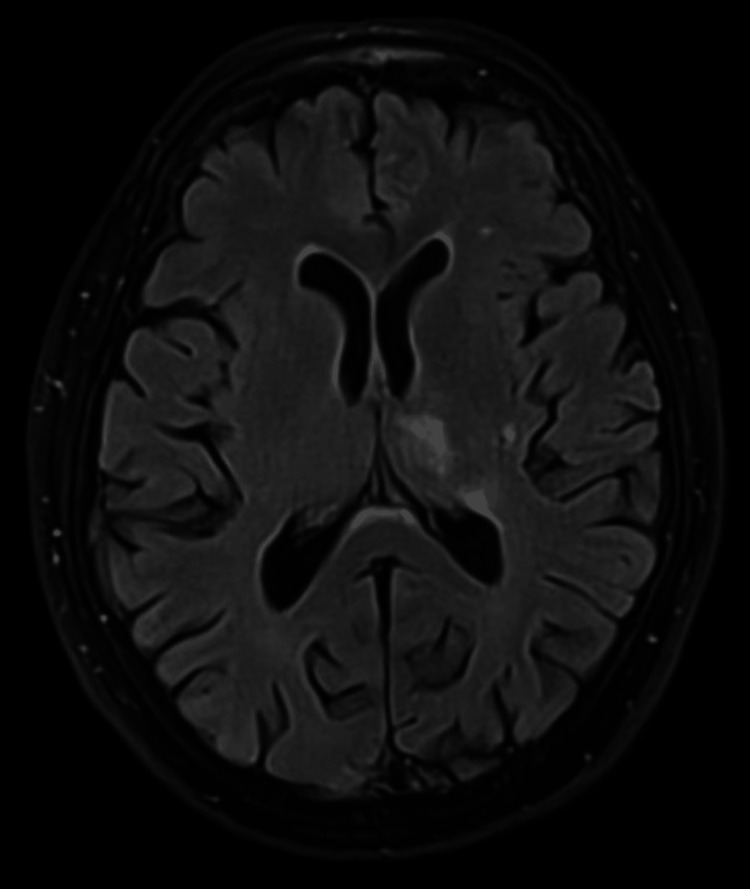
Magnetic resonance imaging showing acute infarct involving the posterior limb of the left internal capsule, extending into the medial temporal lobe and caudate nucleus

The initial differential diagnosis included delirium secondary to infection or metabolic disturbance, such as acute kidney injury or posterior circulation stroke. However, laboratory investigations, including full blood count, electrolytes, and inflammatory markers, were unremarkable except for a mildly elevated serum calcium of 2.62 mmol/L (normal range: 2.2-2.6 mmol/L). Non-contrast computed tomography (CT) of the head imaging showed no acute abnormalities. Due to persistent confusion, a magnetic resonance imaging (MRI) of the brain with diffusion-weighted imaging (DWI) was requested to look for evidence of encephalitis or acute stroke. This revealed an acute infarct involving the posterior limb of the left internal capsule, extending into the medial temporal lobe and caudate nucleus (Figure 2). 

Over 48 hours, her cognitive function improved, scoring 4/4 on repeat 4AT assessments, with improvement in orientation and memory. She was commenced on apixaban and bisoprolol for atrial fibrillation and atorvastatin for secondary prevention. After a multidisciplinary review, she was discharged home with follow-up by local community stroke teams and speech and language therapy.

## Discussion

Lacunar infarcts account for approximately 25% of all ischemic strokes and result from the occlusion of perforating arteries supplying the subcortical structures [3]. The internal capsule is particularly vulnerable to infarction due to the dense concentration of projection fibers and its end-arterial vascular anatomy [4,5]. Infarction within the posterior limb typically results in contralateral motor weakness or isolated sensory loss. However, involvement of adjacent fibers can disrupt thalamocortical and frontostriatal pathways, producing cognitive symptoms without motor deficits [6].

This case illustrates an atypical presentation of internal capsule stroke manifesting as isolated acute confusion. Zhao et al. [7] and Coenen et al. [8] demonstrated that injury to white matter tracts connecting the thalamus, caudate nucleus, and medial temporal lobe correlates with post-stroke cognitive impairment [9,10]. These findings are supported by multicenter studies corroborating that a minor subcortical lesion produces significant neurocognitive sequelae [11].

Acute confusion is a common presentation on the acute medical take for the general physician with a very wide differential diagnosis. While normal CT imaging is reassuring, ruling out important differentials such as acute bleeds or malignancy is of utmost importance. MRI with DWI remains the most sensitive modality for detecting small and acute infarcts. In this case, the absence of focal neurology initially misled clinicians toward a diagnosis of delirium. However, persistent confusion in a patient with atrial fibrillation without other systemic symptoms should raise suspicion for a potential vascular aetiology. 

Recovery of cognition after small deep infarcts varies case by case [12]. Some patients experience complete resolution, while others develop persistent executive dysfunction and apathy due to the disruption of frontosubcortical circuits. Early recognition and secondary prevention are therefore essential.

This case underscores the need for clinicians to have a high index of suspicion in patients presenting with acute confusion for cerebrovascular disease, even in the absence of motor or sensory deficits, and that stroke may present as isolated confusion without typical focal neurological deficits. Correlation of clinical symptoms with neuroanatomical localization is critical in atypical presentations. This case is also a great example displaying the importance of MRI in ambiguous neurologic presentations, as CT is often within normal limits in early ischemic presentations.

## Conclusions

Firstly, our case demonstrates the importance of considering ischemic stroke in patients presenting with acute confusion and no motor or sensory deficits, especially when risk factors are present and no other cause is identified. Secondly, we demonstrate that internal capsule infarction can disrupt strategic thalamocortical and associative pathways, leading to transient cognitive impairment. Finally, prompt MRI with DWI remains essential for the diagnosis and management of stroke.
